# Emerging Issues in Virus Taxonomy

**DOI:** 10.3201/eid1001.030279

**Published:** 2004-01

**Authors:** Marc H.V. van Regenmortel, Brian W.J. Mahy

**Affiliations:** * University of Strasbourg, France; † Centers for Disease Control and Prevention, Atlanta, Georgia, USA

**Keywords:** nature of viruses, virus species, virus identification, typography of virus names, binomial names of virus species

## Abstract

Viruses occupy a unique position in biology. Although they possess some of the properties of living systems such as having a genome, they are actually nonliving infectious entities and should not be considered microorganisms. A clear distinction should be drawn between the terms virus, virion, and virus species. Species is the most fundamental taxonomic category used in all biological classification. In 1991, the International Committee on Taxonomy of Viruses (ICTV) decided that the category of virus species should be used in virus classification together with the categories of genus and family. More than 50 ICTV study groups were given the task of demarcating the 1,550 viral species that were recognized in the 7th ICTV report, which was published in 2000. We briefly describe the changes in virus classification that were introduced in that report. We also discuss recent proposals to introduce a nonlatinized binomial nomenclature for virus species.

In the 7th report of the International Committee on Taxonomy of Viruses (ICTV), viruses were described as elementary biosystems that possess some of the properties of living systems such as having a genome and being able to adapt to a changing environment (*1*). Viruses belong to biology because they possess genes, replicate, evolve, and are adapted to particular hosts, biotic habitats, and ecological niches. However, viruses cannot capture and store free energy, and they are not functionally active outside their host cells. Although they are pathogens, viruses should not be considered pathogenic microorganisms since they are not alive. The simplest system that can be said to be alive is a cell. Cells acquire the autonomy that is characteristic of living systems through a complex set of integrated, metabolic activities. However, none of the individual constituents of cells, such as organelles or macromolecules, can be said to be alive. A virus becomes part of a living system only after it has infected a host cell and its genome becomes integrated with that of the cell. Viruses are replicated only through the metabolic activities of infected cells, and they occupy a unique position in biology. They are nonliving infectious entities that can be said, at best, to lead a kind of borrowed life.

## Viruses versus Virus Particles or Virions

A virus is a general term which denotes any number of concrete objects that possess various relational properties (for instance, its host, vector, and infectivity) that arise by virtue of a relation with other objects. These relational properties, also called emergent properties, are characteristic of the viral biosystem as a whole and are not present in its constituent parts. When a virus undergoes its so-called life cycle, it takes on various forms and manifestations, for instance, as a replicating nucleic acid in the host cell or vector. One stage in this cycle is the virus particle or virion, which is characterized by intrinsic properties such as size, mass, chemical composition, nucleotide sequence of the genome, and amino acid sequence of protein subunits, among others. Virions can be fully described by their intrinsic chemical and physical properties, and that description does not entail the relational properties that belong to the virus.

Confusion sometimes arises when a virion is called the virus, as, for instance, when one refers to “the picture of the virus” or to the process of “purifying the virus.” What is actually meant in such cases is a virus particle, not a virus. Confusing virus with virion is similar to confusing the entity insect, which comprises several different life stages, with a single one of these stages, such as pupa, caterpillar, or butterfly.

### The Species Concept in Virology

Since viruses, like other biological entities, are concrete objects located in time and space, their classification is a purely conceptual construction, based on the use of abstract categories that have no spatiotemporal localization (*1*). Virus classification places the viruses in a series of classes or taxonomic categories with a hierarchical structure, the ranks being the species, genus, family, and order. These classes are abstractions, i.e., conceptual constructions produced by the mind, and they should not be confused with the real, disease-causing objects studied by virologists. Centrifuging the family *Picornaviridae*, the genus *Enterovirus,* or the species *Poliovirus* is impossible for the simple reason that abstractions, i.e. ideas, cannot be centrifuged. For the same reason, a virus species cannot cause a disease, since abstractions do not cause diseases (with the exception of psychosomatic disease). The concrete virus entity that causes a disease can, however, be a member of an abstract virus species. Concrete objects like viruses can be members of an abstract entity, that is, an entity of a different logical type, although they cannot be a part of such an abstract concept. Class inclusion or class membership is the correct relationship between a virus and the species category to which it belongs. One often reads that the species *Mus musculus* has been inoculated with one or other virus species. The correct statement is that a mouse (a member of the species *Mus musculus*) has been inoculated with a member of viral species X.

Although species is the most fundamental taxonomic category in all biological classifications, it was only in 1991 that the ICTV agreed that the concept of virus species should be uniformly applied in virus classification. For many years, plant virologists had been arguing that the concept of species was not applicable to viruses because they are not sexually reproducing organisms (*2*,*3*). These virologists took the view that the only legitimate species concept was that of biological species, defined on the basis of gene pools and reproductive isolation. Such a concept is clearly not applicable to entities like viruses that replicate by clonal means (*4*). However, many other species concepts are currently used in biology, some of them applicable to asexual organisms. As many as 22 different species concepts have been applied in various fields of biology (*5*).

For virus species to become accepted by the virologic community, coining a definition to which virologists could subscribe was necessary. In 1991, the ICTV endorsed the following definition: “A virus species is a polythetic class of viruses that constitute a replicating lineage and occupy a particular ecological niche” (*6*–*8*). This definition was no longer based on purely phenetic criteria of similar characteristics but stressed the cohesive forces present in ancestral-descendant biological populations that share a common biotic niche. Another important feature of the definition is that a virus species is defined as a polythetic class rather than as a traditional universal class. A polythetic class consists of members which have a number of properties in common but which do not all share a single common property that could be used as a defining and discriminating property of the species because it is absent in other species. This situation is illustrated in the Figure.

**Figure:**
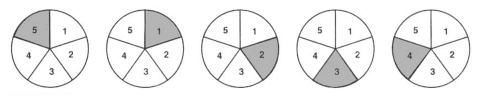
Schematic representation of five members of a polythetic class characterized by five properties, 1–5. Each member possesses several of these properties, but no single property is present in all the members of the class. This missing property in each case is represented by the gray sector.

The advantage of defining virus species as polythetic classes is that individual viruses that lack one or other characteristic normally considered typical of the species can be accommodated. This advantage is particularly relevant for entities like viruses that undergo continual evolutionary changes and show considerable variability. In practice, a single discriminating characteristic, such as a particular host reaction or a certain percentage of genome sequence identity, cannot be a defining property of any virus species. Rather, a combination of properties always provides the rationale for deciding whether a virus should be considered a member of a particular species. Thus, different virus species do not have sharp boundaries. Rather, they should be viewed as fuzzy sets with hazy boundaries (*1*,*9*).

Species are thus very different from the other taxonomic categories used in virus classification such as genera and families. A viral family, for instance, is a so-called universal class that consists of members, all of which share a number of defining properties that are both necessary and sufficient for class membership (*10*). Allocating a virus to a family or a genus is thus an easy task since all that is required is to consider a few morphologic or chemical features that suffice to unambiguously position the virus in the classification scheme. For instance, all members of the family *Herpesviridae* are enveloped viruses that contain an icosahedral particle and double-stranded DNA, whereas all members of the family *Adenoviridae* are nonenveloped viruses that contain an icosahedral particle and double-stranded DNA, with projecting fibers at the vertices of the protein shell. In contrast, allocating a virus to a particular species is often a matter of convenience or convention rather than of logical necessity based on an unequivocal defining property.

### Demarcating Virus Species and Identifying Viruses

 It is a common misperception that once the concept of virus species has been defined, deciding if a particular virus is a member of a certain virus species is easy. This expectation arises because of a failure to appreciate that definitions apply only to abstract concepts, such as the notion of species taken as a class. Individual viruses, like individual people or any other concrete entities, can be named and identified by so-called diagnostic properties, but they cannot be defined (*11*). The difference between definition and identification can be illustrated by the following analogy. Transportation vehicles can be classified into categories such as buses, trucks, and cars. Cars can be defined as a type of vehicle with four wheels, capable of transporting a limited number of persons, not exceeding a certain size or weight. However, such a definition will be of no use in discriminating between a Ford and a Toyota. To ascertain whether an individual vehicle corresponds to a particular make of car, a set of distinguishing characteristics that make it possible to identify each car must be used. In a similar way, the theoretical definition of the species category that the ICTV endorsed in 1991 is not helpful for recognizing and distinguishing the viruses that are members of individual species. What is required is that virologists reach an agreement about which diagnostic properties are the most useful for identifying the individual members of a virus species. Since ICTV study groups (*12*) are mostly responsible for deciding which virus species should be recognized within individual genera and families, these specialty groups, with their in-depth knowledge of particular areas in virology, have been given the task of establishing which diagnostic properties are most useful for species demarcation.

To differentiate between individual species, it is necessary to rely on properties that are not present in all the members of a genus or family, since obviously such properties will not permit species demarcation. For example, characteristics such as virion morphology, genome organization, method of replication, and number and size of structural and nonstructural proteins are properties shared by all the members of a genus or family. Therefore, these characteristics cannot be used for demarcating individual species within a given genus. The following properties are useful for discriminating between virus species (*13*): genome sequence relatedness, natural host range, cell and tissue tropism, pathogenicity and cytopathology, mode of transmission, physicochemical properties, and antigenic properties.

 All of these characteristics are not equally important for demarcating species in different viral genera and families, however. There is, in fact, no need to harmonize diagnostic criteria across all species, genera, and families. In some families, certain diagnostic criteria will be more important than in others, not the least because the practical needs for making certain distinctions are not the same in all areas of virology. The major purpose of virus classification is to partition the world of viruses into a coherent scheme of easily recognizable entities that answers to the everyday needs of practicing virologists. From a human perspective, not all hosts are equally relevant. Thus, human pathogens or pathogens that infect animals and plants of economic importance will be studied more intensively than, say, the viruses that infect the myriad species of insects. Finer distinctions based on relatively minor differences in host range, pathogenicity, or antigenicity may thus be made in the case of viruses that are of particular interest to humans. For instance, differences in the antigenic and genomic properties of individual human adenoviruses may be considered sufficient reason to allocate these viruses to separate species, whereas the same degree of antigenic dissimilarity would in other cases lead such entities to be considered serotypes of the same species. Allocating viruses to different species requires that an answer be given to the perplexing question of identity: how different must two viruses be to be considered different types of virus and therefore members of different species? Mutants or pathogenic variants that are clearly distinguishable from the wild-type virus will, however, generally be recognized as being the same type of virus, and they will, therefore, be considered, in terms of taxonomy, to be members of the same virus species.

Deciding whether individual virus isolates correspond to strains or serotypes of one species or belong to separate species remains in many cases one of the challenges that must still be addressed by many ICTV study groups. Virus identification is usually a comparative process whereby individual isolates are compared with the members of established virus species. Since virus species are polythetic, the comparison should involve a number of different characteristics rather than the presence or absence of a single key feature. However, the use of several characteristics is essential only for demarcating individual polythetic species and for constructing an acceptable classification scheme. Once a species has been established on the basis of several demarcation criteria, identifying a virus isolate as a member of that species by considering only a few properties may be possible. For instance, if a virus isolate reacts with a panel of monoclonal antibodies in the same way as an established member of a given species, the virus will be considered as a member of that species. The 7th ICTV Report

The 7th ICTV report (*14*) was published in 2000, five years after the 6th report (*15*). Whereas the 6th report described 1 order, 50 families, and 164 genera, the 7th report contained 3 orders, 63 families, and 240 genera. In the 6th report, >3,600 viral entities were listed, in many cases without a clear indication of their status as species, strains, serotypes, or isolates. In the 7th report, the criteria used for demarcating virus species within a genus were defined for many of the genera, which resulted in a list of 1,550 officially recognized viral species. The major changes in the classification scheme introduced in the 7th report have been summarized by Fauquet and Mayo (*16*).

### Names and Typography of Virus Species

In earlier ICTV reports, names of orders, families, subfamilies, and genera were written in italics with a capital initial letter and had the following endings: -*virales* for orders, -*viridae* for families, -*virinae* for subfamilies and –*virus* for genera. The revised code (*17*) extends this typographic convention to the names of virus species in order to give a visible sign that species were recognized viral taxa, just as are genera and families. In most cases, the English common names of viruses have become the species names, and these are written in italics with the initial letter capitalized (*17*,*18*). The effect is to discriminate between virus species officially recognized by the ICTV and other viral entities such as tentative species, viral strains, serotypes, or other subspecific entities within a species. This new typography has met with some criticism (*19*,*20*) and corresponding rebuttals (*21*,*22*), but it is now generally applied in most scientific journals and books on virology (*23*).

The value of using italics is that it visibly reinforces the status of the corresponding species as a taxonomic entity, i.e., a formal, abstract class, distinct from the concrete viral objects that replicate and cause disease and that are written in Roman characters. Only if it is necessary to draw attention to the taxonomic position of the virus under study will it be necessary to refer to the official species name written in italics. Even then, the official name need be given only once, probably in the introduction or Materials and Methods sections (e.g., *Measles virus****,*** genus *Morbillivirus*, family *Paramyxoviridae*). In publications written in languages other than English, the use of italics for the English official species name would also indicate the alien nature of the term. In such publications, the common names of viruses will be those used in that language and not the English names. The use of italicized English instead of italicized Latin for the names of virus species reflects the emergence of English as the modern language of international scientific communication, and it also does away with the invidious task of having to coin new Latin names for all virus species (*21*).

By introducing italicized virus species names, the ICTV in no way intended to replace the existing vernacular or common names of viruses written in Roman characters (*21*,*24*). The viruses studied by virologists are concrete, disease-causing entities and not abstract classes, and they should continue to be referred to by their common, nonitalicized names. As recently reiterated by Drebot et al. (*25*), only the names of viral taxonomic classes are written in italics, not the names of viruses. In scientific articles, authors need to refer most of the time to the virus as a physical entity rather than as a member of a taxonomic class. Therefore, the common name written in Roman characters will most often be used; the species name, in italics, will appear only once for the purpose of taxonomic placement of the virus being discussed.

### A Proposed Binomial Nomenclature for Virus Species

 For many years, some plant virologists have been using an unofficial binomial system for referring to virus species (as well as to viruses). In this system, the italicized word *virus* appearing at the end of the current official species name is replaced by the genus name, which also ends in “-*virus*” (*20*,*25*). Thus *Bluetongue virus* becomes *Bluetongue orbivirus* and *Measles virus* becomes *Measles morbillivirus*. The advantage of such a system is that inclusion of the genus name in the species name indicates relationships with other viruses and therefore provides additional information about the properties of the members of the species. To nonspecialists, it would then be immediately obvious that Hepatitis A, B, and C viruses are very different entities, belonging to different genera, were their official names *Hepatitis A hepatovirus*, *Hepatitis B* *orthohepadnavirus,* and *Hepatitis C hepacivirus.*

Such a binomial system for species names would also have the advantage of clearly distinguishing between the species name written in italics (*Measles morbillivirus*) and the common, nonitalicized virus name, measles virus. At present, the distinction between the species name and the virus name in most cases relies only on typography (i.e., *Measles virus* versus measles virus), which can lead to confusion (*24*).

Whether nonlatinized binomials should become the official species names of viruses has been debated within the ICTV for many years (*21*,*22*,*25*–*28*). Although most plant virologists have favored the use of binomials for many years (*29*), to what extent human and animal virologists would find the system acceptable has not been known. As the ICTV strives to develop a universal system of nomenclature approved by all virologists (*17*), it is bound to move cautiously before changing all the current, official names of virus species. Since very few virologists express their views on matters of taxonomy (*21*,*22*), successive ICTV Executive Committees have always found it difficult to poll the representative opinion of virologists worldwide (*30*), and it is not clear what sort of democratic process would satisfy those who criticize ICTV decisions. During 2002, efforts were made to canvass virologists regarding their acceptance of a binomial system of species names; the results of two ballots showed that a sizeable majority (80%-85%) of the 250 virologists who expressed an opinion were in favor of a binomial system (*24*,*31*). The new ICTV Executive Committee established at the 12th International Congress of Virology, held in Paris in July 2002, will decide in the near future if binomial names of virus species should be introduced. A list of current virus species names, together with their binomial equivalents, can be found on ICTV net (available from: URL: www.danforthcenter.org/ILTAB/ICTVnet/).

### Abbreviations for Virus Names

 To avoid repetition, authors of virology papers use abbreviations for virus names, once the full name has been given. Since it is only the common names that are used repeatedly in a given text, abbreviating them (rather than the current official species names or their binomial counterparts if binomials were to become the official names) makes sense.

Although the ICTV does not have a constitutional responsibility for devising appropriate abbreviations, it has over the years published several lists of recommended abbreviations of virus names. Initially, these were abbreviations for the common names of viruses (*32*,*33*), but subsequently they were published as abbreviations for the names of virus species (*34*,*35*). Although the names of the viruses and of the corresponding viral species are usually the same, they are not necessarily so, and it could be argued that species names do not need to be abbreviated at all. The abbreviations recommended by ICTV should therefore apply only to the names of viruses. Although an emerging discipline, virus taxonomy is essential to the working virologist, and we need to achieve universal agreement on the principles so that we can freely communicate without misunderstanding (36, *37*).
